# Repair of bone defects with prefabricated vascularized bone grafts and double-labeled bone marrow-derived mesenchymal stem cells in a rat model

**DOI:** 10.1038/srep39431

**Published:** 2017-02-02

**Authors:** Xiao-Rui Jiang, Hui-Ying Yang, Xin-Xin Zhang, Guo-Dong Lin, Yong-Chun Meng, Pei-Xun Zhang, Shan Jiang, Chun-Lei Zhang, Fei Huang, Lin Xu

**Affiliations:** 1 Department of Orthopedics, The Affiliated Yantai Yuhuangding Hospital of Qingdao University, Yantai 264000, P.R. China; 2 Department of Intensive Care Unit, Yantai Infectious Disease Hospital, Yantai 264000, P.R. China; 3 Department of Orthopedics, Canner Hospital, Chinese Academy of Medical Sciences, Peking Union Medical College, Beijing 100000, P.R. China; 4 Department of Orthopedics, The Affiliated Yantai Hospital of Binzhou Medical University, Yantai 264000, P.R. China; 5 Department of Trauma and Orthopedics, Peking University People’s Hospital, Beijing 100000, P.R. China; 6 Southern Medical University, Guangzhou 510515, P.R. China; 7 Binzhou Medical University, Yantai 264000, P.R. China

## Abstract

This study aims to investigate the repair of bone defects with prefabricated vascularized bone grafts and double-labeled bone marrow-derived mesenchymal stem cells (BMSCs) in a rat model. BMSCs were separated from rat bone marrow. LTR-CMVpro-RFP and LTR-CMVpro-GFP were transfected into the BMSCs for *in vitro* and *in vivo* tracking. BMSCs-RFP and BMSCs-GFP were induced into endothelial progenitor cells (EPCs) and osteoblasts (OBs). Rats were divided into five groups: Group A: *in vitro* prefabrication with EPCs-RFP + *in vivo* prefabrication with arteriovenous vascular bundle + secondary OBs-GFP implantation; Group B: *in vitro* prefabrication with EPCs-RFP + secondary OBs-GFP implantation; Group C: *in vivo* prefabrication with arteriovenous vascular bundle + secondary OBs-GFP implantation; Group D: implantation of EPCs-RFP + implantation of with arteriovenous vascular bundle + simultaneous OBs-GFP implantation; Group E: demineralized bone matrix (DBM) grafts (blank control). Among five groups, Group A had the fastest bone regeneration and repair, and the regenerated bone highly resembled normal bone tissues; Group D also had fast bone repair, but the repair was slightly slower than Group A. Therefore, *in vitro* prefabrication with EPCs-RFP plus *in vivo* prefabrication with arteriovenous vascular bundle and secondary OBs-GFP implantation could be the best treatment for bone defect.

Bone defects are usually repaired by implantation of autogenous bone, allogeneic bone and heterogeneous bone^1^,^2^,^3^. Unfortunately, the clinical application of these methods is limited by the risk of inducing extra trauma, the need of a supply source and immunogenicity^4^. In this context, bone tissue engineering has emerged as a new therapy for bone repair using osteoconductive and osteoinductive grafts and autogenous seed cells for the repair of extensive bone defects^5^. Recent studies have provided enriched methods and techniques for bone tissue engineering^6^,^7^,^8^. However, current methods and techniques are limited in their applications, and they fail to meet the clinical requirements regarding the quantity and quality of bone regeneration. An important reason for this is believed to be necrosis of the seed osteoblasts in grafts, which further impairs osteogenic efficiency. Thus, a key requirement for the success of bone tissue engineering is ensuring the early survival of seed cells.

The success of bone tissue engineering lies in ontogenesis and vascularization^9^,^10^. Vessels can transport oxygen and nutrients for cell growth and discharge metabolites. It has been observed that osteoblasts can only survive up to 100 μm from vessels^11^. Fast vascularization in grafts is of great importance for osteogenesis in bone tissue engineering^12^. Therefore, we believed that the low osteogenesis efficiency may be caused by a slow *in vivo* vascularization rate at the early stage of implantation, resulting in shortages of oxygen and nutrients that are required for the survival and proliferation of seed osteoblasts^13^. On these grounds, fast vascularization and a rich vessel net in grafts may be a good solution to the above problem^11^. *In vivo* prefabrication for vascularization has been widely used in bone tissue engineering^14^. However, the seed vascular cells also require nutrients for growth at early stages, and therefore, we propose the *in vitro* prefabrication of vessels. Evidence has demonstrated that vessels prefabricated on grafts before implantation can fuse and connect with autologous vessels after implantation^15^. Researchers have also successfully prefabricated vessels by pre-seeding endothelial progenitor cell-derived endothelial cells on grafts before implantation^16^. In the present study, we aim to investigate the repair of bone defects with prefabricated vascularized bone grafts and double-labeled BMSCs in a rat model.

## Materials and Methods

### Ethic statement

This study was conducted in strict accordance with the recommendations in the Guide for the Care and Use of Laboratory Animals of the National Institutes of Health. The protocol was approved by the Institutional Animal Care and Use Committee of the Affiliated Yantai Hospital of Binzhou Medical University.

### Study subjects

Sprague Dawley (SD) (n = 48; 3 months old; 350 g ± 50 g) rats were purchased from the Department of Animal Science of the Affiliated Yantai Hospital of Binzhou Medical University. Among the 48 rats, 24 were selected for *in vivo* pre-vascularization and carefully numbered for the following observations.

### Isolation and labeling of bone marrow-derived mesenchymal stem cells (BMSCs)

BMSCs were separated from whole marrow from the femurs and tibias of rats and then incubated to the third generation in medium containing 10% fetal bovine serum (FBS) and low-sugar Dulbecco’s modified Eagle medium (DMEM). The third-generation lentiviral vectors, LTR-CMVpro-RFP and LTR-CMVpro-GFP, were constructed and used to prepare red fluorescent protein (RFP)-recombinant lentiviruses and green fluorescent protein (GFP)-recombinant lentiviruses. Following measurement of virus titers, RFP- and GFP-recombinant lentiviruses were transfected into BMSCs *in vitro*. After transfection, BMSCs were labeled with RFP or GFP. As the fluorescent expression approached 100%, the cells were evaluated for their phenotype, proliferation and differentiation.

### Induction and identification of endothelial progenitor cells (EPCs)

In high-sugar and 2% FBS-containing DMEM (EGM-2 SingleQuots), BMSCs were induced into EPCs-RFP, which were incubated at 37 °C with 5% CO_2_ and saturated humidity). After 3 days of induction, the medium was totally renewed, and the suspending cells were removed while the remainder continued to be incubated. Six days later, most cell colonies fused together; part of them were digested with 0.3% pancreatin and centrifuged for cell counting using a hemacytometer, and part of them remained under incubation until the 10^th^ day.

The digested cells in the above experiment were re-suspended in EP tubes (10^6^ cells/tube). EP tubes were divided into two groups: the experimental tube contained FITC-CD133 (10 μL) and PE-VEGFR-2 antibody (10 μL), and the negative control tube contained isotype antibodies, FITC IgG (10 μL) and PE IgG (10 μL). The cells were then incubated in darkness (4 °C, 30 min) and centrifuged (300 g, 5 minutes), followed by removal of the supernatant and rinse. Later, cells were resuspended in paraformaldehyde (10 g/L), stimulated with argon lasers (488 nm) and detected using a flow cytometer. CellQuest software was used to calculate the ratio of positive red/green fluorescence-labeled cells. The actual ratio of positive red/green fluorescence-labeled cells (%) = (ratio of positive red/green fluorescence-labeled cells)_the experimental tube_ − (ratio of positive red/green fluorescence-labeled cells)_the experimental tube_.

Immunohistochemical staining of von Willebrand factor (vWF): On the 6^th^ and 10^th^ days of incubation, once the cells adhered to the glass slide, the cells were rinsed with phosphate buffered saline (PBS) (3 times) and fixed with 4% paraformaldehyde at 4 °C. According to the instructions of the immunohistochemistry kit, vWF was counterstained using rat-anti-vWF primary antibody and biotin labeled goat-anti-rat IgG secondary antibody (vWF antibody, Abcam Company, UK).

### Induction and identification of osteoblasts (OBs)

BMSCs-GFP were seeded in a culture bottle (100 mL). At a cell confluence of 80%, the culture solution was removed. Six mL osteogenic medium (containing 10% FBS, 10 nM dexamethasone, 50 μg/mL VitC, 10 β-glycerophosphate and high-sugar DMEME) were added and incubated at 37 °C with 5% CO_2_ and saturated humidity. The medium was renewed every two days or three days.

Immunohistochemical staining of bone calcium elements: 7 days after induction of osteoblasts, BMSCs-GFP were rinsed with PBS (3 times), fixed (95% alcohol; 20 minutes), and incubated to eliminate the activity of endogenous peroxidase (3% hydrogen peroxide; 5~10 minutes). Next, the cells were rinsed with distilled water (2 min × 3 times) and then blocked with 5% normal goat serum (room temperature; 20 minutes). Following removal of superfluous liquid, the cells were incubated with 50 μL of rabbit-anti-bone calcium element monoclonal antibody (37 °C, 1 hour; 4 °C, overnight). After rinsing with PBS (0.01 M; 3 minutes × 3 times), the cells were incubated with 50 μL of biotinylated goat-anti-rabbit in IgG (37 °C, 1 hour) and again rinsed with 0.01 M PBS (3 minutes × 3 times) for subsequent incubation (37 °C, 1 minutes) with 50 μL of streptavidin-biotinperoxidase complex (SABC). After incubation, the cells were rinsed with PBS (3 minutes × 3 times) and then colored (room temperature, 3~10 minutes) in 100 μL of diaminobenzidine (DAB). The time of coloration depended on the real-time observation of coloration, and the coloration was ended with distilled water, followed by counterstaining with hematoxylin, dehydration, hyalinization and filtering.

Immunohistochemical staining of Alizarin Red-S (ARS): Upon the appearance of round and oval nodules among the BMSCs-GFP, the coverslip was removed, and the cells were rinsed with PBS (3 times) followed by fixation with 75% alcohol (30 minutes), rinsing with distilled water (5 minutes × 3 times) and staining with 2% ARS (2~5 minutes). Subsequently, cells were dehydrated using alcohol gradients, dehydrated with xylene, mounted with resinene and paragraphed.

Immunohistochemical staining of tetracycline fluorescence: Upon the appearance of round and oval nodules among the BMSCs-GFP, the coverslip was removed, and the cells underwent induction first with 0.1 mg/ml tetracycline-containing induction medium (30 minutes) and then with complete induction medium (30 minutes). After the coverslip was removed, the cells were rinsed with PBS (3 times) and then fixed with 75% alcohol (30 minutes) and paragraphed under a fluorescence microscope.

### Efficiency in vascularization *in vitro* and *in vivo*

Demineralized bone matrix (DBM) grafts were prepared in accordance with the method proposed by Tang *et al*.^17^. For *in vitro* prefabrication, EPCs-RFP suspensions (containing 2 × 10^6^ cells) were inoculated into similarly sized DBM grafts (ф 4 mm × 8 mm). Next, the DBM grafts were placed in induction medium with endothelial cells. To determine the best timing for *in vitro* prefabrication, the grafts were incubated for 3 days, 7 days, 10 days and 14 days (3 grafts/time). At the end of incubation, the grafts were fixed with 4% paraformaldehyde and frozen in sections to protect the fluorescent protein. The results of the cytoskeleton and EPCs-RFP combination cultures were observed and compared under a scanning electron microscope. Because the best timing was determined, prefabrication was performed again. Similarly sized grafts (ф 4 mm × 8 mm) and SD rats were prepared. The rats were anesthetized by intraperitoneal injection of 3% Nembutal, and microsurgical techniques were employed to dissociate the femoral artery and vein bundle at the groins for *in vivo* prefabrication. For *in vivo* prefabrication, the grafts were prefabricated and then embedded under the groins, and the artery and vein bundle were implanted into the side channels of the grafts, followed by wrapping with absorbable guided bone regeneration (GBR) membrane or silica membrane. For the prefabrication regime, the grafts were divided into four groups: Group A, Group B, Group C and Group D. Group A received *in vitro* prefabrication + *in vivo* prefabrication, Group B received the *in vitro* prefabrication alone, Group B received the *in vivo* prefabrication alone, and Group D received only the DBM grafts embedded under the rats’ groins (blank control). At 3 days, 7 days. 14 days and 21 days after prefabrication, the grafts were fixed, embedded and divided into sections (3 rats/group/timing). After hematoxylin (HE) staining, the vascular structures of the sections were observed (co-constructed by EPCs-RFP and autologous cell). The micro-vessels at different times were counted. The time point when a group had the largest number of micro-vessels was defined as the best time for *in vitro* prefabrication + *in vivo* prefabrication.

### Evaluation of *in vivo* osteogenesis

After removal of the GBR membrane and silica membrane, well-prefabricated grafts were injected with OBs-GFP-collagen gel suspension (2 × 10^6^ cells) using a microsyringe. Blank grafts received inoculations of EPCs-RFP or OBs-GFP-collagen suspensions and were implanted with a femur arteriovenous beam at the bone defect. Using microsurgical techniques, 7-mm-long bone and periosteum defects were made in SD rats. Next, the pre-cultured grafts were implanted and fixed with four-well bone plates (25 mm) and self-taping screws. A blank control group was implanted with only blank DBM grafts. Altogether, there were five groups of rats: Group A received *in vitro* prefabrication with EPCs-RFP, *in vivo* prefabrication and secondary OBs-GFP implantation; Group B received *in vitro* prefabrication with EPCs-RFP and secondary OBs-GFP implantation; Group C received *in vivo* prefabrication and secondary OBs-GFP implantation; Group D received implantation of EPCs-RFP, implantation of a femur arteriovenous beam and simultaneous OBs-GFP implantation; and Group E received only DBM grafts (blank control). At the 4^th^, 8^th^ and 12^th^ weeks, we recorded vascularization and osteogenesis (3 rats/group/timing). All rats were numbered, and the grafts were implanted into the same rat in which they were preimplanted in the bond defect model.

### Pathological and radiological observations

General condition and anatomical observations were recorded. We observed post-operative activity, feeding and wound healing, inspected osteotylus and insertion status at the broken end of the bone through the original incision after killing the rats and we tracked the fluorescence using a laser scanning confocal microscope. Before the rats were killed, an X-ray photograph (45 kV, 100 mA, 0.08 s, and focal distance of 90 cm) was taken to observe bone healing in the hind limbs. Using the X-ray photographs, bone formation at the bone graft area was leveled by the Lane-sandhu method^18^. After the rats were killed, the graft was taken out, and the two interfaces were removed following decalcification and dehydration with sodium formate and embedding with paraffin. Cuts were made at 1/4 the length of the graft from the two interfaces and the center such that the graft was cut into 4 equally sized slices for HE staining. For the determination of osteogenesis performance and vascularization, Mapinfo software was used to calculate the area percentage of new bone and the cross-sectional area ratio of the vessels.

### Hematoxylin-eosin (HE) staining

MVD is a quantitative index of vascularization. The MVD was measured using the method proposed by Weidner *et al*.^19^. After HE staining, vessel-rich sections were selected for calculating the MVD using a low-power lens (×100). Three fields were randomly selected to observe the MVD (×200), and the MVD was calculated as the mean value of 3 measurements. For a reliable calculation, bleeding areas, fibering areas and fringe areas were excluded from vessel counting. MVD was expressedas the mean ± standard deviation.

### Quantitative real-time polymerase chain reaction (qRT-PCR)

At the 4^th^, 8^th^ and 12^th^ weeks after surgery, the rats were killed, and expressions of osteocalcin (OCN) and vascular endothelial growth factor (VEGF) were detected by RT-PCR. The total RNA was extracted using a Trizol kit, and RNA concentration and purity were determined using an ultraviolet spectrometry photometer. Using agarose gel electrophoresis (AGE), RNA integrity was measured. A Primescript^TM^ RT reagent kit (TaKaRa Biotechnology (Dalian) Co., Ltd) was used for reverse transcription. An SYBR^®^ premix Ex Taq^TM^ kit was used for PCR amplification. The primers are listed in Table 1. The internal reference was GAPDH. The ratios of the gray value (target band/internal reference) at the 4^th^, 8^th^ and 12^th^ weeks were recorded and compared based on a variance analysis.

### Biomechanical test

At the 12^th^ week, the grafts of all groups were subjected to compression strain in addition to two normal rats (3 months old) via a three-point bending test. The radius was taken out as a whole, followed by removal of cartilage and fixation of the proximal and distal radii using high-strength dental base acrylic resin powder. The proximal and distal radii were fixed at the same level. After 20 minutes of fixation, the diaphysis was covered with wet gauze and was placed on a multi-function mechanical test for measuring peak load (F) and bending stress (δ) upon uniformbend loading at 1 mm/minute and a span of 4 cm. δ = 8FL/πd^3^, where L was the fixed distance (40 mm), and d was the external diameter at the bone split.

### Statistical analysis

All the data are expressed as the mean ± standard deviation and analyzed using SPSS 21.0 software. The measured data showed a normal distribution, and between-group comparisons were conducted using t tests, within-group comparisons were tested with a one-way analysis of variance (ANOVA) (after homogeneity test of variances was performed), and between-group comparisons among groups and comparisons of means were performed using least significant difference tests. *P* < 0.05 was considered statistically significant.

## Results

### EPCs phenotype

**Figure 1:**
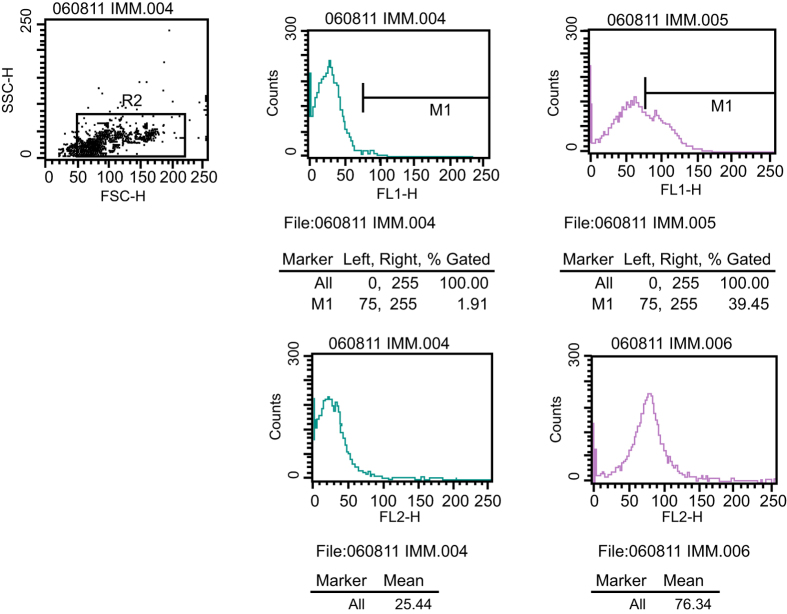
Positive expressions of VEGFR-2 and CD133. Note: VEFGR-2, vascular endothelial growth factor receptor 2.

The cell surface markers, VEGFR-2 and CD 133, were examined using a flow cytometer. VEGFR-2 is a specific marker of endotheliocytes that presents high expression in both EPCs and endotheliocytes of mature vessels. CD133 is a specific marker in hematopoietic stem cells, which gradually disappears as EPC differentiate into endotheliocytes in mature vessels. As presented in Fig. 1 (supplementary Table 1), VEGFR-2 expression was (52.4 ± 6.2)% and CD133 expression was (34.1 ± 5.9)%. Thus, the detected cells were considered EPCs.

### The expression of von Willebrand factor (vWF) in EPCs

**Figure 2:**
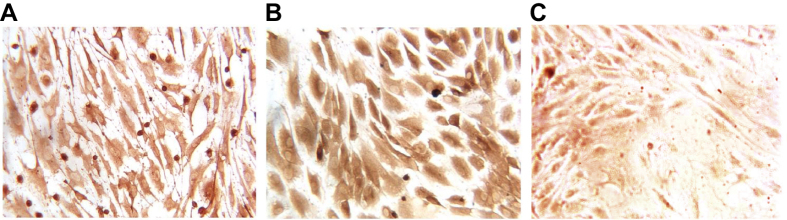
Immunohistochemistry (IHC) of EPCs. Note: (**A**) positive vWF expression at the 6^th^ day of incubation; (**B**) positive vWF expression at the 10^th^ day; (**C**) positive vWF expression at in the negative control; EPCs, endothelial progenitor cells; vWF, von Willebrand factor.

VWF is a specific surface marker of vascular endothelial cells. At the 6^th^ day of staining, cells began to present positive vWF expression, and the vWF positive expression was obviously increased at the 10^th^ day (Fig. 2).

### Identification of OBs

**Figure 3:**
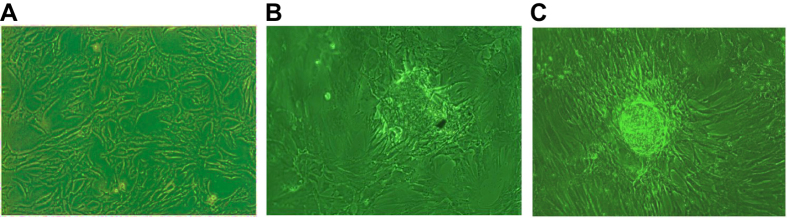
Morphology of osteoblasts induced with BM-BMSCs (×100). Note: (**A**) morphology of osteoblasts induced with BM-BMSCs at the early stage of induction; (**B**) morphology of osteoblasts induced with BM-BMSCs at the 7^th^ day of induction; (**C**) morphology of osteoblasts induced with BM-BMSCs at the 12–14^th^ days of induction; BM-BMSCs, bone marrow-derived mesenchymal stem cells.

BMSCs in osteoblast induction presented a slow growth rate and irregular shapes, such as triangles and polygons. However, the number of cells markedly increased, and some of them grew into multiple layers, such as osteoblasts (Fig. 3A). Cells fused together and formed calcium crystals by the 7^th^ day of induction (Fig. 3B) and grew into calcium nodules (Fig. 3C).

**Figure 4:**
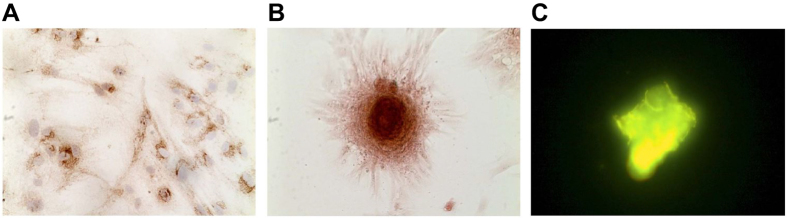
Identification of induced osteoblasts using immunohistochemical staining. Note: (**A**) immunohistochemical staining with bone calcium elements; (**B**) immunohistochemical staining with Alizanrin Red-s; (**C**) immunohistochemical staining with tetracycline.

As presented in Fig. 4A, after immunohistochemical staining of bone calcium elements, induced cells turned brown with positive expression of bone calcium element. By the 15^th^ day of induction, after performing immunohistochemical staining of ARS, the cells aggregated to mineralized nodules, an orange complex of ARS and calcium salt (Fig. 4B). After immunohistochemical staining for tetracycline fluorescence, the induced cells formed calcium nodules, which were stained golden yellow with tetracycline fluorescence, and cells were radially arranged around the nodules. The un-induced cells propagated, presenting contact inhibition and no overlapping growth or calcium nodules (Fig. 4C).

### Comparisons of efficiency in vascularization among five groups

**Figure 5:**
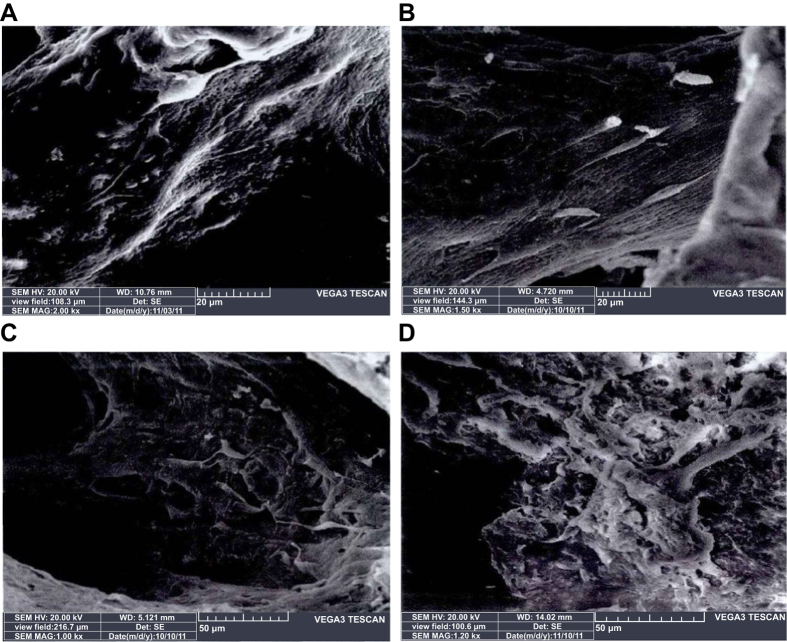
*In vitro* co-culture of EPCs-RFP and DBM grafts among five groups. Note: (**A**) *in vitro* co-culture of EPCs-RFP and DBM grafts at the 3^rd^ day (×2000); (**B**) *in vitro* co-culture of EPCs-RFP and DBM grafts at the 7^th^ day (×1500); (**C**) *in vitro* co-culture of EPCs-RFP and DBM grafts at the 10^th^ day (×1000); (**D**) *in vitro* co-culture of EPCs-RFP and DBM grafts at the 14^th^ day (×1200).

On the 3^th^, 7^th^, 10^th^ and 14^th^ days of *in vitro* incubation of EPCs-RFP in DBM grafts, cell attachment was observed. As shown in Fig. 5 (supplementary Table 2), the cells mainly attached to the holes and to the surface of the grafts with their rivet-like pseudopods. On the 10^th^ and 14^th^ days, the cells exhibited a multi-layered state, interacting with each other like a net. This suggested that DBM had a high cellular affinity. Additionally, using an electron microscope, significantly more attached EPCs-RFP were observed on the 10^th^ (36.42 ± 5.32) and 14^th^ days (37.04 ± 4.28) compared to on the 7^th^ (22.56 ± 4.45) and 3^rd^ days (10.21 ± 3.87) (all *P* < 0.05). However, no difference was identified between the number of EPCs-RFPs on the 10^th^ and 14^th^ days (*P* > 0.05). Therefore, the 10^th^ day was considered as the best time point for *in vitro* prefabrication. As presented in Table 2, in Group A, the growth of micro-vessels slowed by the 14^th^ day, and no statistically significant increase in the number of micro-vessels was observed on the 21^st^ day compared with that on the 14^th^ day (*P* > 0.05). Group A had significantly higher MVD than Group B, Group C and the blank control group (all *P* < 0.05). Groups B and C, although exhibiting slower growth than Group A, showed no significant reduction in growth during the 21 days. Thus, we believed that day 14 was best time point for the combination of *in vitro* and *in vivo* prefabrication.

### Comparisons of general information and bone regeneration among five groups

All of the included rats regained their energy and appetites by 3 days after the operation and regained their daily movement by 5 days after the operation. No rats had any complication, as infection or cutaneous necrosis. At 4 weeks after operation, we observed the bone defect. In the blank control group, the bone defects were found to be filled with the undecomposed graft observable by the naked eye. In Group A, the bone tissue regenerated, and the regenerated bone undistinguishably resembled normal bone tissue. In Groups B and C, the bone defects were filed with thin bone tissue and some decomposed graft. In Group D, the bone defects were filled with compact regenerated bone, whereas the outline of the bone defect was still recognizable. In Group E, the bone defects were filled with thin bone tissue and had visible depression, remarkably different from normal bone tissue. At 8 weeks after operation, in Group A, the bone defects were basically recovered; in Groups B, and C, the bond defects still exhibited a good recovery rate, and the regenerated bone was similar to normal tissues; in Group D, the bone defects regenerated new bone, and the regenerated bone was observable by the naked eye; in Group E, the bone defects exhibited very low recovery speed and seldom recovered. At the 12^th^ week, Groups A, B, C and D had totally recovered from their bone defects. Nevertheless, in Group E, the bone defects remained unrecovered, and only part of the graft was decomposed.

### Comparisons of X-ray scanning score among five groups

X-ray scanning results corroborated the anatomical observations. At 4 weeks after operation, Group A had bone defects that were completely filled by regenerated tissue, and cortical bone had regenerated at the bone defects. Groups B and C had bone defects that were mostly filled by regenerated bone. Group D had bone defects completely filled, but the regenerated bone layer was relatively thin compared with Group A. Group E had bone defects mostly filled by undecomposed grafts and little regenerated bone. At 8 weeks after operation, the bone defects in Group A were completely repaired; those in Group B, C and D were completely filled with regenerated bone, and the cortical bone in those groups was also regenerated. The bone defects in Group E exhibited slow bone regeneration, and the grafts were seldom absorbed. At the 12^th^ week, the regenerated bone in Groups A, B, C and D were equivalent to normal bone, and the bone defects exhibited good recovery, while Group E presented no significant improvements compared with the 8^th^ week.

The X-ray scans of the five groups were graded (Table 3). Groups B and C presented no significant differences for all time points (*P* > 0.05). However, at all time points, the relative trends of the scores of the five groups decreased in the following order: A > D > B ≈ C > E, and all the comparisons were statistically significant (all *P* < 0.05).

### Comparisons of ratio of vascular area and new bone formation among five groups

As observed by the tissue sections, Group E did not exhibit osteoblasts until the 4^th^ week and had a long osteogenesis period. At the endpoint of our observations (the 12^th^ week), Group E still exhibited osteogenesis, with osteons sparsely distributed, bone lamella out of alignment, bone trabecula having visible osteoblasts, cavum medullare carrying a few mesenchymal cells and large cavum medullare formation failing.

**Figure 6:**
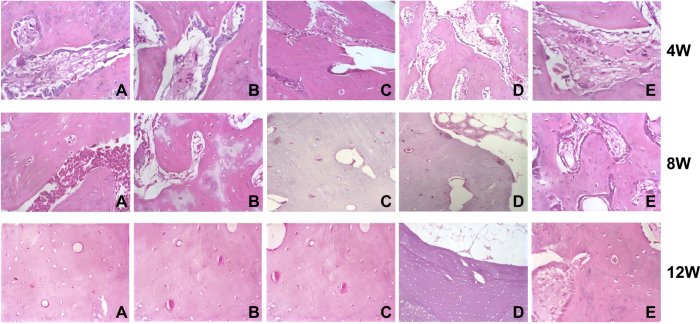
Histological observation of osteogenesis among the five groups at the 4^th^, 8^th^ and 12^th^ weeks. Note: (**A**) *in vitro* prefabrication with EPCs-RFP + *in vivo* prefabrication with arteriovenous vascular bundle + secondary osteoblast (OB)-GFP implantation; (**B**) *in vitro* prefabrication with EPCs-RFP + secondary OBs-GFP implantation; (**C**) *in vivo* prefabrication with arteriovenous vascular bundle + secondary OBs-GFP implantation; (**D**) implantation of EPCs-RFP + implantation of with arteriovenous vascular bundle + simultaneous OBs-GFP implantation; (**E**) only DBM grafts (blank control); ECs, endothelial cells; OB, osteoblast; RFP, red fluorescence protein; GFP, green fluorescence protein.

At the 4^th^ week after operation, Groups A, B, C and D presented obvious signs of osteogenesis. Moreover, Group A exhibited not only end-to-center osteogenesis but also osteogenesis spreading from the implantation site of the vein beam. Groups B and C presented no significant differences in any indicators at all time points. Group A and D, although presenting no difference in vascular area, had significantly different bone regeneration areas (A > D, *P* < 0.05). The bone regeneration area decreased in the following order: A > D > B ≈ C > E (all *P* < 0.05) (Fig. 6). Judging from the bone regeneration area and the vascular area, we found that Group A had the strongest efficiency in vascularization and osteogenesis (Table 3).

### Comparison of MVD among five groups

According to the method proposed by Weidner *et al*., MVD was calculated (Table 4). At the 4^th^ week, the MVDs of Groups A, B, C and D were significantly higher than Group E (all *P* < 0.05), while no such difference was observed between Groups A/D and Groups B/C and D (all *P* > 0.05) (A ≈ D > B ≈ C > E). At the 8^th^ week, the MVDs of the five groups followed the same sequence as that at the 4^th^ week and significantly increased (*P* < 0.05). At the 12^th^ week, the MVDs in Group E were still significantly lower than the other four groups (*P* < 0.05), and the other four groups showed no significant differences in MVD (*P* > 0.05). Compared with the MVD at the 8^th^ week, the MVD of Groups B, C and D continued to increase (all *P* < 0.05), while the MVD of Group E presented no significant increase (*P* > 0.05). These results suggested that the *in vitro* prefabrication + *in vivo* prefabrication groups (Group A and Group D) had significantly faster increases in MVD (or faster vascularization) from the initial time point when compared with the *in vitro* prefabrication (Group B) or *in vivo* prefabrication groups (Group C). Groups B and C did not catch up with Groups A and D until the 8^th^ week.

### Comparisons of OCN and VEGF mRNA expressions among five groups

At the 4^th^ week after operation, Groups B, C and E had significantly lower OCN and VEGF mRNA expressions than Groups A and D, and OCN and VEGF mRNA expressions in Group E were significantly lower than in Groups B and C (all *P* < 0.05). At the 8^th^ and 12^th^ weeks, Groups A, B, C and D had significantly higher OCN and VEGF mRNA expressions than Group E (all *P* < 0.05), while Groups B and C had slightly lower OCN and VEGF mRNA expressions than Groups A and D at the 8^th^ week, although not statistically significant, and they showed no difference from Groups A and D at the 12^th^ week (all *P* > 0.05) (Table 5).

### Comparisons of the maximum load and the torsional strength among five groups

At 12^th^ week after operation, the two biomechanical indicators in Groups B and C were significantly lower compared with Groups A and D, and the indicators in Group E were remarkably lower than the other four groups (all *P* < 0.05). Group A and Group D exhibited no significant differences in the two biomechanical indicators compared to normal rats (*P* > 0.05). As shown in Table 6, the regenerated bone in the groups that received *in vitro* prefabrication + *in vivo* prefabrication (Group A and Group D) had the most similar biomechanical performance to normal bone tissue.

## Discussion

The present study was designed to investigate the repair of bone defects with prefabricated vascularized bone grafts and fluorescent double labeling of bone marrow-derived mesenchymal stem cells in a rat model. We believed that a well-prefabricated vessel net in the grafts before implantation may aid in the proliferation and survival of osteoblasts and, therefore, promote the quality and quantity of bone regeneration.

In our study, we developed a co-culture system, inducing EPCs and osteoblasts from the same BMSCs. In agreement with previous studies, our results suggested that BMSCs were suitable for the induction of seed cells in bone tissue engineering. BMSCs are adult stem cells, capable of self-renewal, high hyperplasia and differentiation into multiple cells. Prior to our study, many studies had reported good osteogenic and differentiation potentials and activity of BMSCs^20^,^21^,^22^.

As observed, the induced EPCs were found to have high vascularization efficiency. Consistently, in previous studies, EPCs were also reported to be effective in stimulating vascularization and vascular repair in tumorigenesis and cardiovascular diseases^23^,^24^. In our study, we found that the *in vitro* prefabrication + *in vivo* prefabrication group reached its best efficiency at 14^th^ day, which was significantly shorter than the *in vitro* prefabrication-only group (group b) and the *in vivo* prefabrication-only (Group c). Consistently, Ruchi Mishra *et al*. also reported that vessels continued to increase until the 3^rd^ week of *in vivo* prefabrication^25^. The mechanism for this difference should be further studied in future work. A possible explanation may be that the *in vitro* culture of EPCs ensures the transition to endothelial colony-forming cells (ECFCs). As observed, the EPCs had positive expression of vWF at the 6^th^ day of culture, an indication that EPCs had developed into mature endothelial cells^26^. These ECFCs had high proliferation activity, promoting fast vascularization^27^,^28^.

After implantation of the grafts, we observed a much faster vascularization and higher MVD in both Group A and Group D. This result further validated that *in vitro* prefabrication + *in vivo* prefabrication had better efficiency for vascularization. Group A and Group D had better and quicker bone repair. Based on these results, we believed that vitro prefabrication + *in vivo* prefabrication for vascularization in bone tissue engineering promotes osteogenic efficiency through improving vascularization. However, a slight difference between Group A and Group D was identified in osteogenic efficiency. By X-ray scanning and histological observations, we found that Group A had a larger osteogenic density and osteogenic area at the 4^th^ week after operation. This difference may result in the time difference between vascularization and osteogenesis. This time difference allowed Group A begin developing vessels earlier than Group D and therefore to supply of the nutrients and oxygen required for the growth of osteoblasts early in the first 4^th^ weeks. Taken together, we proposed that *in vitro* prefabrication with EPCs-RFP + *in vivo* prefabrication + secondary OBs-GFP implantation may be a promising method of bone regeneration in bone tissue engineering.

In conclusion, we provide direct evidence that *In vitro* prefabrication with EPCs-RFP plus secondary OBs-GFP implantation of *in vivo* prefabrication with arteriovenous vascular bundle could be the best treatment for bone defect. To validate these results, more comprehensive clinical observations are needed in the future.

## Additional Information

**How to cite this article:** Jiang, X.-R. *et al*. Repair of bone defects with prefabricated vascularized bone grafts and double-labeled bone marrow-derived mesenchymal stem cells in a rat model. *Sci. Rep.* **7**, 39431; doi: 10.1038/srep39431 (2017).

**Publisher's note:** Springer Nature remains neutral with regard to jurisdictional claims in published maps and institutional affiliations.

## Supplementary Material

Supplementary Information

Supplementary Dataset

## Figures and Tables

**Table 1:** Primer sequences for qRT-PCR.

Gene	Primer sequence (5′-3′)	Product size
OCN	F: GAATCCCGCAAAGGTG	185 bp
	R: GCTGACCACATTGGCTT	
VEGF	F: AAGTCCTGGAGCGTTCCCTGT	168 bp
	R: GACAAGCCCAGGCGGTGA	
GAPDH	F: AAGGTCGGAGTCAACGGATTT	372 bp
	R: GGCATCAGCAGAAGGAGCAG	

Note: OCN, osteocalcin; VEGF, vascular endothelial growth factor, qRT-PCR, quantitative real-time polymerase chain reaction.

**Table 2:** Comparison of MVD at different time points among five groups.

Grouping	3 d	7 d	14 d	21 d
Group a	2.58 ± 0.72	6.83 ± 1.16	9.35 ± 1.58	9.40 ± 1.52
Group b	1.31 ± 0.54	4.28 ± 1.02*	6.11 ± 1.34*	7.24 ± 1.46*
Group c	1.64 ± 0.61	4.57 ± 1.21*	6.23 ± 1.55*	7.57 ± 1.37*
Group d	0.69 ± 0.12*	1.76 ± 0.52*	2.31 ± 0.84*	2.84 ± 0.97*

Note: MVD, microvessel density; *P < 0.05, in comparison with Group a.

**Table 3:** The X-ray scanning score, ratio of vascular area and new bone formation at different time points among five groups.

Sample grouping	4^th^ week	8^th^ week	12^th^ week
Group A			
X-ray scanning score	4.62 ± 0.51	7.08 ± 0.73	9.83 ± 0.56
Ratio of vascular area ratio	6.38 ± 0.24	8.54 ± 0.21	12.11 ± 0.16
Ratio of new bone formation	5.15 ± 0.14	12.64 ± 0.13	18.17 ± 0.51
Group B			
X-ray scanning score	3.36 ± 0.86	5.79 ± 0.45^#^	7.86 ± 0.76^#^
Ratio of vascular area ratio	3.24 ± 0.12^#^	5.87 ± 0.14^#^	8.16 ± 0.32^#^
Ratio of new bone formation	1.52 ± 0.16^#^	4.28 ± 0.31^#^	6.89 ± 0.72^#^
Group C			
X-ray scanning score	3.25 ± 0.87	5.82 ± 0.43	7.79 ± 0.66^#^
Ratio of vascular area ratio	3.32 ± 0.14^#^	5.88 ± 0.16^#^	8.11 ± 0.31^#^
Ratio of new bone formation	1.50 ± 0.13^#^	4.19 ± 0.32^#^	6.87 ± 0.68^#^
Group D			
X-ray scanning score	3.59 ± 0.96	6.53 ± 0.55	8.98 ± 0.79
Ratio of vascular area ratio	6.24 ± 0.22	8.42 ± 0.15	12.16 ± 0.52
Ratio of new bone formation	1.78 ± 0.26^#^	7.02 ± 0.41^#^	13.15 ± 0.81^#^
Group E			
X-ray scanning score	1.72 ± 0.57^#^	2.47 ± 0.45^#^	3.89 ± 0.63^#^
Ratio of vascular area ratio	0.25 ± 0.02^#^	0.81 ± 0.12^#^	2.04 ± 0.04^#^
Ratio of new bone formation	0.12 ± 0.01^#^	0.47 ± 0.04^#^	0.79 ± 0.07^#^

Note: ^#^*P* < 0.05, in comparison with Group A.

**Table 4:** Comparisons of the mean MVDs at different time points among five groups.

Group	4^th^ week	8^th^ week	12^th^ week
Group A	12.62 ± 0.69	14.57 ± 1.21	16.61 ± 1.26
Group B	5.02 ± 1.08^#^	12.10 ± 1.14	16.43 ± 1.29
Group C	5.08 ± 1.10^#^	12.13 ± 1.19	16.57 ± 1.33
Group D	12.32 ± 1.38	14.51 ± 1.25	16.63 ± 1.34
Group E	2.92 ± 1.03^#^	8.13 ± 1.14^#^	8.28 ± 0.92^#^

Note: ^#^*P* < 0.05, in comparison with Group A.

**Table 5:** Comparisons of OCN and VEGF mRNA expressions at different time points among five groups.

Group		OCN mRNA expression	VEGF mRNA expression
Group A			
	4^th^	0.85 ± 0.11	0.79 ± 0.11
	8^th^	1.02 ± 0.13	1.01 ± 0.05
	12^th^	1.33 ± 0.11	1.36 ± 0.11
Group B			
	4^th^	0.63 ± 0.07^#^	0.61 ± 0.06^#^
	8^th^	0.89 ± 0.12	0.94 ± 0.09
	12^th^	1.12 ± 0.10	1.34 ± 0.12
Group C			
	4^th^	0.64 ± 0.06^#^	0.62 ± 0.05^#^
	8^th^	0.90 ± 0.12	0.95 ± 0.11
	12^th^	1.14 ± 0.09	1.35 ± 0.09
Group D			
	4^th^	0.63 ± 0.12^#^	0.81 ± 0.08
	8^th^	0.95 ± 0.15	0.98 ± 0.09
	12^th^	1.32 ± 0.13	1.35 ± 0.14
Group E			
	4^th^	0.41 ± 0.06^#^	0.48 ± 0.04^#^
	8^th^	0.53 ± 0.09^#^	0.56 ± 0.06^#^
	12^th^	0.58 ± 0.10^#^	0.57 ± 0.05^#^

Note: ^#^*P* < 0.05, in comparison with Group A.

**Table 6:** Comparisons of the maximum load and the torsional strength at different time points among five groups.

Group	12^th^ week
Group A	
Maximum load (N)	176.31 ± 9.84
Torsional strength (Mpa)	4.65 ± 0.12
Group B	
Maximum load (N)	149.45 ± 4.02^#^
Torsional strength (Mpa)	4.23 ± 0.14^#^
Group C	
Maximum load (N)	148.86 ± 4.39^#^
Torsional strength (Mpa)	4.29 ± 0.13^#^
Group D	
Maximum load (N)	174.86 ± 9.79
Torsional strength (Mpa)	4.68 ± 0.13
Group E	
Maximum load (N)	105.56 ± 3.62^#^
Torsional strength (Mpa)	1.26 ± 0.03^#^
Group F	
Maximum load (N)	178.10 ± 2.37
Torsional strength (Mpa)	4.72 ± 0.14

Note: ^#^*P* < 0.05, in comparison with normal rats.
