# Author Correction: Repair of bone defects with prefabricated vascularized bone grafts and double-labeled bone marrow-derived mesenchymal stem cells in a rat model

**DOI:** 10.1038/s41598-020-69955-3

**Published:** 2020-07-30

**Authors:** Xiao-Rui Jiang, Hui-Ying Yang, Xin-Xin Zhang, Guo-Dong Lin, Yong-Chun Meng, Pei-Xun Zhang, Shan Jiang, Chun-Lei Zhang, Fei Huang, Lin Xu

**Affiliations:** 1 Department of Orthopedics, The Affiliated Yantai Yuhuangding Hospital of Qingdao University, Yantai, 264000 P.R. China; 2 Department of Intensive Care Unit, Yantai Infectious Disease Hospital, Yantai, 264000 P.R. China; 3 Department of Orthopedics, Canner Hospital, Chinese Academy of Medical Sciences, Peking Union Medical College, Beijing, 100000 P.R. China; 4 Department of Orthopedics, The Affiliated Yantai Hospital of Binzhou Medical University, Yantai, 264000 P.R. China; 5 Department of Trauma and Orthopedics, Peking University People’s Hospital, Beijing, 100000 P.R. China; 6 Southern Medical University, Guangzhou, 510515 P.R. China; 7 Binzhou Medical University, Yantai, 264000 P.R. China

Correction to: *Scientific Reports* https://doi.org/10.1038/srep39431, published online 02 February 2017

In Figure 6, the image of 12W for group C is incorrect. The correct Figure 6 appears below as Figure 1.

**Figure 1:**
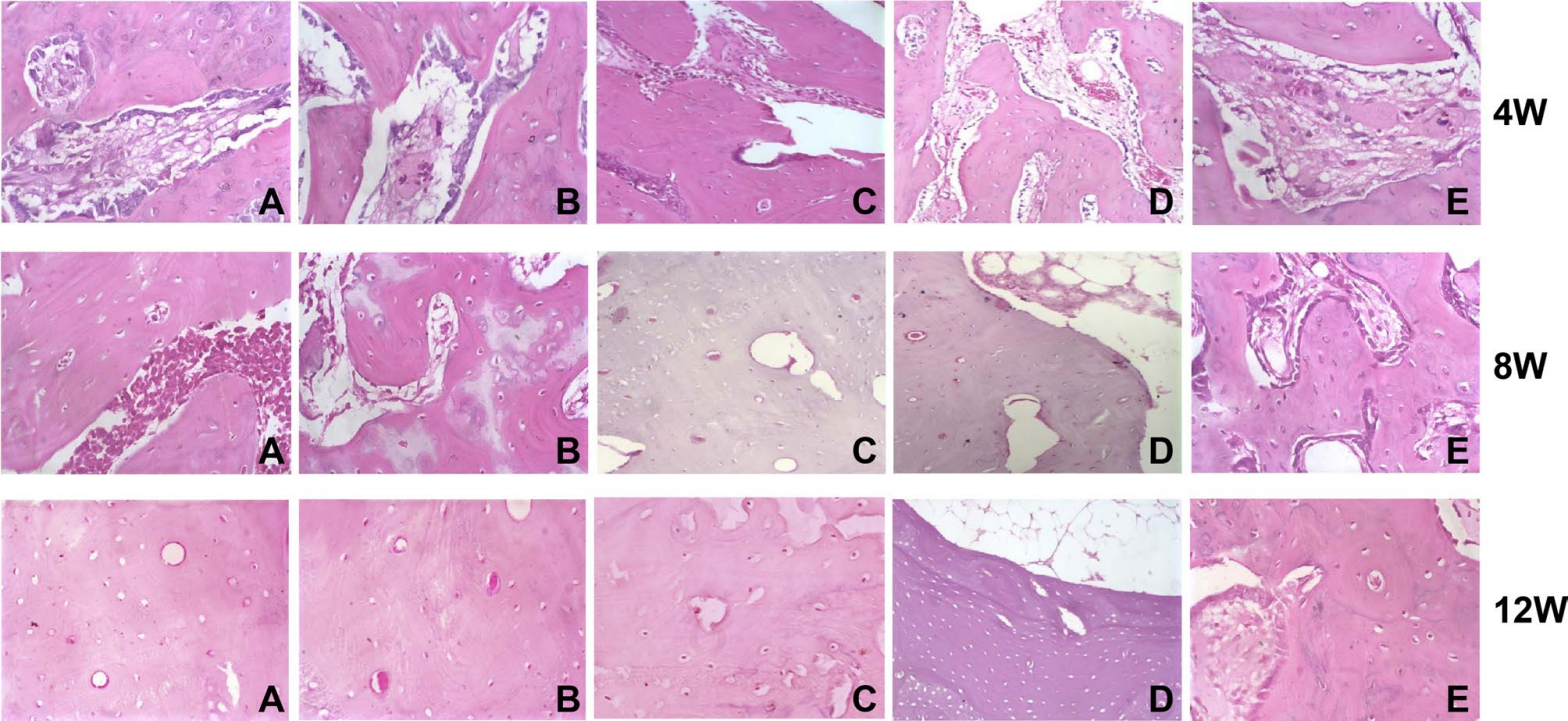
.

