# Supplementary material for: Repair of bone defects with prefabricated vascularized bone grafts and double-labeled bone marrow-derived mesenchymal stem cells in a rat model
Source: Sci Rep. 2017 Feb 2;7:39431. doi: 10.1038/srep39431 (PMC5288698; doi:10.1038/srep39431)
Supplement: Supplementary Information [file srep39431-s1.pdf]

Repair of bone defects with prefabricated vascularized bone grafts and double-labeled bone marrow-derived mesenchymal stem cells in a rat model

Running title: Prefabricated vascularized grafts in bone repair

Xiao-Rui Jiang 1, #, Hui-Ying Yang 2, #, Xin-Xin Zhang 3, #, Guo-Dong Lin 1, Yong-Chun Meng 4, Pei-Xun Zhang 5, Shan Jiang 6, Chun-Lei Zhang 7, Fei Huang 7, Lin Xu 4,*

1 Department of Orthopedics, The Affiliated Yantai Yuhuangding Hospital of Qingdao University, Yantai 264000, P.R. China

2 Department of Intensive Care Unit, Yantai Infectious Disease Hospital, Yantai 264000, P.R. China

3 Department of Orthopedics, Canner Hospital, Chinese Academy of Medical Sciences, Peking Union Medical College, Beijing 100000, P.R. China

4 Department of Orthopedics, The Affiliated Yantai Hospital of Binzhou Medical University, Yantai 264000, P.R. China

5 Department of Trauma and Orthopedics, Peking University People's Hospital, Beijing 100000, P.R. China

6 Southern Medical University, Guangzhou 510515, P.R. China

7 Binzhou Medical University, Yantai 264000, P.R. China

# These are regarded as co-first authors.

* Correspondence to: Dr. Lin Xu, Department of Orthopedics, The Affiliated Yantai Hospital of Binzhou Medical University, No. 717, Jinbu Street, Mouping District, Yantai 264000, Shandong Province, P.R. China

Email: dheh_ab@163.com

Tel: +86-0535-2123456

Acknowledgments

This study was supported by a program for Yantai Science and Technology Development (No. 2011208, 2011209) and projects from the Shandong Natural Science Foundation (Y2008C18), Shandong Science and Technology Development (2011QW034), The National Natural Science Fund (No. 31271284, 81301570, 31070866), The Educational Ministry New Century Excellent Talents Support Project (No. BMU20110270), and the Chinese National Ministry of Science and Technology 973 Project Planning (No. 2014CB542200). We would also like to thank all participants enrolled in the present study.

Figure 1 Positive cell number of BMSCs after induction

VEGFR-2	CD133
45.6	38.7
57.7	36.2
53.9	27.4

Figure 5 Attached EPCs on DBM grafts

3d	7d	10d	14d
13.52	27.14	37.93	32.49
11.15	22.28	40.81	37.64
5.96	18.26	32.39	40.99

Table 2

	MVD			
	a	b	c	d
3 days	1.8	1.79	0.98	0.8
	3.22	0.72	1.76	0.71
	2.72	1.42	2.18	0.56
7 days	5.57	5.18	3.26	2.22

	7.86	3.17	4.81	1.86
	7.06	4.48	5.64	1.2
14 days	7.64	7.3	4.55	3.05
	10.75	4.66	6.54	2.48
	9.66	6.37	7.6	1.4
21 days	7.75	8.53	6.08	3.7
	10.75	5.66	7.84	3.03
	9.7	7.53	8.78	1.79

Table 3

	X ray score						Rat
	A	B	C	D	E		A
4w	4.07	4.12	2.31	4.44	1.83	4w	6.12
	5.07	2.43	3.42	3.78	2.23		6.59
	4.72	3.53	4.02	2.55	1.1		6.43
8w	6.29	6.19	5.35	7.02	2.56	8w	8.31
	7.73	5.3	5.9	6.64	2.87		8.73
	7.22	5.88	6.2	5.93	1.98		8.58
12w	9.22	8.53	7.07	9.68	4.01	12w	11.94
	10.33	7.04	7.92	9.14	4.45		12.25
	9.94	8.01	8.38	8.12	3.21		12.14

Table 4 Mean MVDs at different time points

	A	B	C	D	E
4w	11.87	3.85	3.89	10.82	1.8
	13.23	5.98	6.06	13.54	3.83
	12.76	5.23	5.3	12.59	3.12
8w	13.26	10.86	10.84	13.16	6.89
	15.64	13.11	13.19	15.62	9.14
	14.81	12.32	12.36	14.76	8.35
12w	15.24	15.03	15.13	15.18	7.28
	17.73	17.57	17.75	17.82	9.1
	16.86	16.68	16.83	16.89	8.46

Table 5 *OCN* mRNA

	A	B	C	D	E		A
4w	0.73	0.55	0.57	0.5	0.34	4w	0.67
	0.95	0.69	0.69	0.74	0.46		0.89
	0.87	0.64	0.65	0.65	0.42		0.81
8w	0.88	0.76	0.77	0.79	0.43	8w	0.96
	1.14	1	1.01	1.08	0.61		1.05
	1.05	0.91	0.92	0.98	0.55		1.02
12w	1.21	1.01	1.04	1.18	0.47	12w	1.24
	1.43	1.21	1.22	1.44	0.67		1.46
3	1.35	1.14	1.16	1.35	0.6		1.38

Table 6

Maximum load (N)						To	
A	B	C	D	E	Normal	A	B
165.64	146.46	145.41	164.25	101.64	175.53	4.52	4.08

185.03	154.02	153.79	183.54	108.77	180.2	4.76	4.35
178.25	147.88	147.36	176.79	106.27	178.57	4.67	4.26

io of vascular area ratio

B	C	D	E
3.11	3.17	6	0.23
3.35	3.44	6.44	0.27
3.26	3.35	6.28	0.25
5.72	5.71	8.26	0.68
5.99	6.02	8.55	0.92
5.9	5.91	8.45	0.83
7.81	7.77	11.6	2
8.44	8.38	12.62	2.08
8.22	8.17	12.26	2.05

Ratio of new bone formation

	A	B	C	D	E
4w	5	1.35	1.36	1.5	0.11
	5.27	1.66	1.62	2.01	0.13
	5.18	1.55	1.53	1.83	0.12
8w	12.5	3.94	3.84	6.58	0.43
	12.76	4.55	4.47	7.38	0.51
	12.67	4.34	4.25	7.1	0.48
12w	17.62	6.11	6.13	12.27	0.71
	18.62	7.53	7.47	13.87	0.85
	18.27	7.03	7	13.31	0.8

VEGF mRNA

B	C	D	E
0.54	0.57	0.72	0.44
0.66	0.66	0.88	0.52
0.62	0.63	0.83	0.49
0.84	0.83	0.88	0.51
1.02	1.05	1.06	0.6
0.96	0.97	1	0.57
1.21	1.25	1.2	0.52
1.45	1.43	1.47	0.61
1.36	1.37	1.38	0.58

orsional strength (Mpa)

C	D	E	Normal
4.15	4.54	1.23	4.57

4.41	4.8	1.29	4.84
4.32	4.71	1.27	4.75
